# Informal Mathematical Thinking: Invariance of the Role of Domain-General and Domain-Specific Precursors in Spain and Chile

**DOI:** 10.3390/jintelligence13100128

**Published:** 2025-10-08

**Authors:** Gamal Cerda, Carlos Pérez, Eugenio Chandía, Estíbaliz Aragón, José I. Navarro

**Affiliations:** 1 Department of Research Methodology and Educational Informatics, University of Concepción, Concepción 4030000, Chile; gamal.cerda@udec.cl; 2 Department of Curriculum and Instruction, University of Concepción, Concepción 4030000, Chile; echandia@udec.cl; 3 Department of Psychology, University of Cádiz, 11519 Cadiz, Spain; estivaliz.aragon@uca.es (E.A.); jose.navarro@uca.es (J.I.N.)

**Keywords:** early numeracy, symbolic comparison, cognitive predictors, preschool education, cross-cultural study, structural equation modeling, domain-general skills, domain-specific skills

## Abstract

This study examines how domain-general (processing speed and receptive vocabulary) and domain-specific (symbolic and non-symbolic comparison) cognitive skills contribute to early informal mathematical thinking in preschoolers. The aim was to assess the invariance of these predictive relationships across two sociocultural contexts: Chilean and Spanish samples. A total of 130 children participated, and structural equation modeling was used to estimate latent structures and test multigroup invariance. The results revealed a consistent latent structure across samples and a significant contribution of symbolic and non-symbolic comparison to early math performance, while processing speed and vocabulary showed context-specific variations. These findings indicate that although foundational mathematical competencies rely on common cognitive mechanisms, cultural and educational contexts modulate the strength of these associations. This study contributes to understanding the cognitive architecture underlying early numeracy and highlights the importance of culturally sensitive assessment and intervention strategies.

## 1. Introduction

When children enter the school system, they already possess informal mathematical knowledge acquired through interactions with their environment, family members, peers, and exposure to media such as the Internet and television. It is an informal form of knowledge used to solve different problems, as required by their real-life environment (Malaspina Quevedo 2017), a type of mathematical thinking from which it can be inferred that children are able to think and understand certain kinds of mathematical content. An example of such content is additive composition, which enables the child to distinguish and count small sets of objects (Clements and Sarama 2014). Informal knowledge serves as a basis for interpreting the formal mathematics that they will learn in school and for progressing toward building more complex notions and skills. It is also a kind of preconceptual structure that exerts significant impact on the meaningful learning of school mathematics (Purpura et al. 2013). This takes on particular relevance, given that mathematical knowledge at this early stage provides a significant foundation over which other academic skills can be built. These early mathematical skills are among the most powerful predictors of future academic success in the discipline (Devlin et al. 2022). Mathematical performance is a basic instrumental competence, not just for meeting the demands of the educational system, but also for responding to life’s challenges. However, it is estimated that a considerable percentage of students encounter learning difficulties in this subject (Mammarella et al. 2021).

Before they enter the formal school system, children attending early childhood education present a broad range of individual differences with regard to knowledge of early numeracy. These differences probably originate from diverse experiences in their family environment and sociocultural context (Muñez et al. 2021). Difficulties usually arise early on and continue over time (OECD 2023). Nevertheless, the levels of cognitive development traversed by students seem to be universal in nature and exhibit a discernible pattern (Ong 2016).

From this perspective, the importance and presence are recognized by the so-called domain-general and domain-specific precursors (Bernal-Ruiz and Cerda 2024). These precursors take on special importance in mathematical cognition research, since it was shown that their early detection helps to reduce the risk that difficulties in mathematics may arise in future (Costa et al. 2018). Domain-general precursors are cognitive factors that are not only present in mathematics, but in all types of learning as well, such as general intelligence, working memory, processing speed, and cognitive flexibility. They are considered higher-order functions because they allow for the ordering and direction of the totality of cognitive and behavioral operations (Diamond 2020). Processing speed and receptive vocabulary have shown particular relevance in early mathematical development.

Processing speed has a positive impact on mathematical cognition (Formoso et al. 2018), with specific processing speed being able to predict mathematical performance and generally contributing to academic achievement in elementary school (Cheng et al. 2022). Vocabulary, due to its symbolic and representative function, is essential for the development of language and numerical knowledge, with a close link between numerical knowledge and vocabulary evident in its predictive power vis-à-vis mathematical performance (Zhang 2016).

Domain-specific precursors are understood as abilities that are predictive of specific mathematical learning. In the case of mathematics, these abilities include counting, numerical estimation, symbolic and non-symbolic comparisons (Aragón et al. 2019). The comparison of dot matrices and Arabic numerals, especially the latter, is used to identify students with problems in learning mathematics such as dyscalculia (Bugden et al. 2021), or who are at risk of presenting math learning difficulties (Bull et al. 2021). Research on both general and specific precursors has largely yielded multivariate models that show the effect that precursors have on mathematical competence through their distinct interrelationships (Barnes et al. 2020). The accumulation of evidence confirms that both domain-general and domain-specific precursors consistently influence subsequent mathematical performance across diverse evaluated contexts (Peres Nogues and Vargas Dorneles 2021; Träff et al. 2020).

The role of sociocultural context in mathematical development cannot be understated. Socioeconomic differences exist from the first years of schooling between students from different groups, manifesting as achievement gaps (Mackintosh and Rowe 2021). Specialized research endorses both the existence of said socioeconomic gap and its increasing evolutionary effect on the levels of mathematics learning achievement and quality, as schooling progresses. The gap is not limited to any specific scenario but is evident in different parts of the world, including Latin American contexts (Hascoët et al. 2021). In Latin America, socioeconomic segmentation of educational establishments is the primary underlying factor that explains the disparities in mathematical performance evidenced in comparisons between academic institutions (Cervini et al. 2016).

International assessments provide clear evidence of performance differences between countries. Recent TIMSS and PISA studies indicate that Spanish students consistently outperform Chilean students in mathematics assessments (OECD 2023). These differences may stem from variations in early childhood mathematics curriculum emphasis, teacher preparation programs, and family mathematical engagement practices. Such performance gaps highlight the importance of examining whether the cognitive architecture underlying mathematical thinking varies across different educational and cultural contexts.

Despite significant cultural differences due to distinct geographical, historical and social contexts, Chile and Spain share important educational characteristics that make meaningful comparisons possible. Both countries share the Spanish language and have similar primary school mathematics curricula. Spain’s Decree No. 198/2014 and Chile’s Ministry of Education (MINEDUC 2018) guidelines establish comparable learning objectives: by completion of first grade at around age 7, pupils must be able to perform single-digit addition and subtraction, and by third grade, they must be able to use multiplication and division to solve math problems (Pina et al. 2021). Teacher training in both countries exhibits similar characteristics of perceived academic authority (Zamora-Poblete et al. 2020), and initial teacher training programs are similar in duration and design (Muñiz Rodríguez et al. 2016). However, important differences exist in socioeconomic composition of public schools, teacher hiring requirements, and classroom supervision practices that may contribute to observed performance differences.

The theoretical framework guiding this investigation draws from Vygotsky’s sociocultural theory, which sustains that learning is a cultural phenomenon and children from different cultures adopt different learning styles (Das 2020; Saxe et al. 2024). According to this perspective, development occurs as students engage with individuals who have superior knowledge or skills. Mathematical thinking evolves not only through formal educational domains within the school system but also through informal contexts encountered in students’ daily lives. This development occurs as they engage with society, including its culture, language, and traditions. Therefore, formal and informal types of thinking are intricately intertwined throughout one’s life, and this relationship can influence the development of mathematical skills, conceptual understanding, and problem-solving ability. These activities provide groundwork for developing further mathematical skills (Alsina and Berciano 2018), establishing a synergistic dynamic whose principles are embodied in approaches such as realistic mathematics education (Fauziah and Juandi 2022).

This study adopts an exploratory comparative approach to examine whether the cognitive architecture underlying early mathematical thinking demonstrates invariance across different sociocultural contexts. Based on the accumulated evidence and theoretical framework, we sought to analyze the critical and possibly interdependent role of domain-general and domain-specific precursors. We examined whether tasks involving processing speed and receptive vocabulary (as general predictors) and the comparison of symbolic and non-symbolic magnitudes (as domain-specific predictors) presented identical relationships in both the Chilean and Spanish samples, and congruently predicted the explained variance of informal mathematical thinking.

Specifically, the following hypotheses are proposed: H1: There are significant differences in the level of informal mathematical thinking and its four dimensions, with the Spanish sample expected to show higher performance due to contextual differences and early stimulation patterns. H2: A robust structural equation modeling (SEM) framework can identify the interaction between general and domain-specific cognitive precursors, as well as their explanatory contribution to informal mathematical thinking, with potential invariance across both samples.

**Figure 1:**
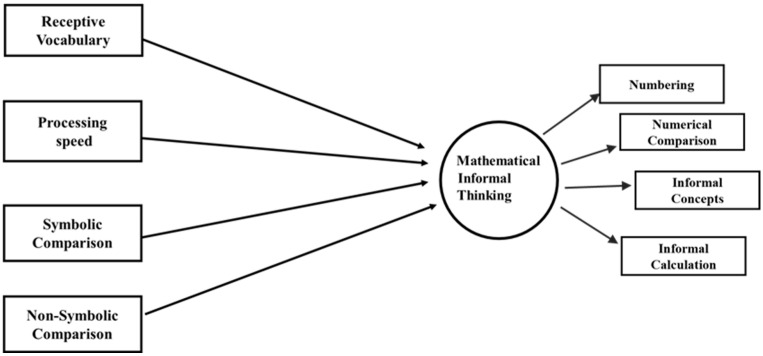
Hypothesized Structural Equation Model of Informal Mathematical Thinking.

Thus, the study objective is to determine the potential differences in levels of informal mathematical thinking between the Chilean sample and Spanish sample, with respect to domain general and domain specific precursors such as receptive vocabulary, processing speed and symbolic and non-symbolic comparison. We propose to achieve this through the examination of an SEM model that postulates the relationship between the various domain-general and domain-specific precursors vis-à-vis informal mathematical thinking, as illustrated in Figure 1. Potential similarities between these models can then be verified across both groups of students. This goal incorporates the sociocultural perspective by Rogoff et al. (2018), suggesting that research should examine and not assume cross-cutting generalities between populations and contexts, and that children’s development should be studied within their native ecosystem.

## 2. Materials and Methods

### 2.1. Participants

The schools were chosen non-probabilistically according to accessibility and considering similar socioeconomic characteristics in order to minimize potential differences in access to complementary private supports. The establishments in Spain were public schools in the community of Andalusia and in Chile they were public nursery schools that are part of the Integra kindergarten network.

On the other hand, in Chile (Valenzuela et al. 2014) as in Spain, the distribution of students between public and private education follows a clear socioeconomic pattern, with most children from high-income families attending private schools (Díez-Gutiérrez and Palomo-Cermeño 2023). In Chile and in Spain, a steady decline in public school enrollment is observed as a result of declining birth rates. This translates into courses having fewer students and charter schools being preferred over public schools (Merchán and Javier 2021). In addition to the above, children with special education needs and students whose families did not authorize their participation in the study were excluded from the cohorts. Table 1 shows the distribution by gender and age of the participants in each country.

### 2.2. Instruments

Mathematical Competence Assessment Test, TEMA-3, Subtest of Informal Thinking Assessment (Ginsburg et al. 2007). This subtest assesses informal mathematical thinking between the ages of 3 and 9 years, in the components of numeration (23 items), comparison of quantities (6 items), informal calculation (8 items) and basic informal concepts (4 items), all of which are ordered based on their difficulty and an average of 15 to 20 min of individual administration. Cronbach’s alpha was 0.92.

Symbolic and Non-Symbolic Comparison Test (Nosworthy et al. 2013). The test evaluates the ability to compare symbolic and non-symbolic magnitudes. In the pencil-paper modality it consists of 56 pairs, both symbolic (digits) and non-symbolic (dots), on printed sheets. The subject must compare the pairs, crossing out the component representing a higher quantity. The correct answers are counted within a maximum of two minutes for each comparison type. Cronbach’s alpha for the symbolic comparison subtest was 0.93, and for the non-symbolic comparison, 0.95.

WPPSI-III Key Test (Wechsler 2009). This test evaluates processing speed along with visual perception, hand-eye coordination, learning ability, cognitive flexibility, and short-term memory. The student must complete each test item with symbols that he or she will choose from a series of five referential models. The task consists of 64 items, 5 of which are examples, and must be completed within a maximum of two minutes. This test is part of the Wechsler Intelligence Scale for preschool and elementary school children. Its Cronbach alpha was 0.86.

The DST-J Dyslexia Screening Test-Junior (Fawcett and Nicolson 2013). This test was used to examine Receptive Vocabulary. It comprises 16 items and a practice image. The evaluator verbalizes the word and the examinee is asked to choose the corresponding image from among four options. The Cronbach alpha was 0.75.

### 2.3. Procedure

First of all, the participating schools were contacted to explain the scope of the research, and obtain the informed consent of the Chilean and Spanish parents/guardians and pupils before starting the fieldwork, with due consideration of the ethical considerations of working with children. Likewise, the assessments were conducted during class hours, not during recess, and the researchers coordinated with the administration so that the evaluations would not disrupt the classes.

To ensure the equi-comparability of the assessment results, the two country teams met for training in a similar test application method and to define a common protocol and a practical instructional guide that was subsequently used by the evaluators in both countries. These measures were also designed to prevent the introduction of any extraneous variables deriving from the evaluation methodology.

In both countries, two sessions of individual assessment were conducted with each participant, each session lasting from 20 to 30 min. In one of these sessions, the assessment tests given were those for receptive vocabulary, processing speed, and symbolic and non-symbolic magnitude comparison. In the other session, the TEMA-3 Subtest of Informal Mathematical Thinking was administered. The order of test administration was random for both the inter- and intra-sessions.

### 2.4. Data Analysis

Initially, a descriptive analysis of the data was conducted, calculating the mean and standard deviation for each variable, both for the overall sample and by country. Welch’s *t*-test was used to detect differences in means between countries, and Cohen’s d was calculated as the effect size.

According to Westland (2010), the minimum sample size for a SEM study depends on the relationship between latent variables and indicators, as well as the ability to detect relationships between latent variables. In the case of the present study, using the formula provided in that article for the relationship between 4 observed variables and 1 latent variable in the measurement model, a minimum of 100 cases is required. For the structural model, for the purposes of sample size calculation, we assumed a restricted model in which all predictor variables are associated with a single latent variable. To test an association model between two latent variables with a minimum correlation of r = 0.3, which corresponds to a moderate relationship, a sample size of 90 cases is needed to achieve 80% power at a 5% significance level. Therefore, the obtained sample size is acceptable for testing this SEM model.

Correlation matrices between variables in Chile and Spain were analyzed using tests for complete matrices and specific correlations proposed by Steiger (1980).

To determine whether the prediction model for informal thinking by general and specific determinants is common or distinct between Chile and Spain, it was considered appropriate calculate the measurement invariance for the informal thinking instrument, and then the predictive invariance of the regression model of determinants on informal thinking measured as a latent variable.

Confirmatory factor analysis was carried out using the lavaan and psych packages in R, version 4, with MLR estimation. Fit indices included the Comparative Fit Index (CFI), the Tucker–Lewis Index (TLI), the Root Mean Square Error of Approximation (RMSEA), and the Standardized Root Mean Square Residual (SRMR). RMSEA values of 0.06 or lower, SRMR of 0.08 or lower, and CFI and TLI values of 0.95 or higher are recognized acceptable (Hu and Bentler 1999). Differences between models were evaluated using the scaled difference chi-square test statistic (Satorra and Bentler 2001).

In this study, an assessment was made of cross-country factorial invariance following Steenkamp and Baumgartner’s (1998) approach, which involves evaluating configural, metric, and scalar invariances. Ensuring these invariances is essential for accurate comparisons of factor means. Initially, each model must demonstrate adequate fit individually. Configural invariance, the baseline model, confirms that factor structures are consistent across groups. Metric invariance, necessary for comparing regression slopes or longitudinal change scores, requires equivalent factorial loadings in all groups. Scalar invariance, crucial for attributing observed item mean differences to the underlying construct differences, mandates equal item intercepts for interval and ratio items across groups. Using the criteria defined by Chen (2007) for adequate sample sizes (N > 300), metric non-invariance should be established for changes in CFI greater than −0.10, along with a change in RMSEA > 0.015 or a change in SRMR > 0.03. Scalar non-invariance must be confirmed for changes in CFI greater than −0.10, along with a change in RMSEA > 0.015 or a change in SRMR > 0.01.

To test predictive invariance, four multigroup SEM structural models were also calculated. In the first model, an analog of configural invariance was tested, with free regression coefficients by country. The second model tested scalar invariance, with equal factor loadings and item intercepts for the countries, while allowing free regression coefficients by country. The third model corresponds to the configural invariance model but with equal regression coefficients by country. Finally, the fourth model considers both scalar measurement invariance and equal coefficients for both countries. In all models, age and gender were included as control variables, with country-specific coefficients. To analyze invariance, both the Chi-Square difference and the fit in CFI and RMSEA were studied, using the criteria applied to evaluate adequate fit in confirmatory factor analysis (Millsap 1998).

Preliminary regression analyses were also conducted to examine the basic relationships between predictors and informal mathematical thinking. While these analyses revealed that the cognitive predictors explained substantial variance in mathematical performance (R^2^ = 0.539 for the combined sample, R^2^ = 0.686 for Chile, and R^2^ = 0.526 for Spain), they also demonstrated important country-specific differences in predictor patterns. Specifically, vocabulary emerged as a significant predictor only in Chile (β = 0.322, *p* < 0.001), while processing speed was significant only in Spain (β = 0.334, *p* < 0.001). These differential patterns across countries, combined with the theoretical importance of modeling informal mathematical thinking as a latent construct composed of multiple indicators, justified the adoption of structural equation modeling (SEM) as our primary analytical approach.

SEM offers methodological advantages over multiple regression by accounting for measurement error, modeling informal mathematical thinking as a latent construct, enabling rigorous invariance testing across countries, and providing comprehensive model fit evaluation. These capabilities are essential for valid cross-cultural conclusions.

To establish the importance of each predictor, both in Spain and Chile, general dominance analysis was performed on the determination coefficients of the latent variable (Budescu and Azen 2004).

Ethical Statements This research adhered to ethical guidelines formulated by psychologists and complied with the legal frameworks established by European and Chilean laws relating to research with children. Before assessments were conducted, written informed consent was obtained from all parents, with a commitment to maintain the confidentiality of the study’s content.

## 3. Results

In accordance with the data of the informal thinking scales, as well as for the general and specific determinants (see Table 2), we can observe that, in all of the variables, except for Vocabulary and Non-symbolic Comparison, statistically significant differences by country are shown, with the highest scores recorded in all the cases for Spain. Moderate differences appeared in keys, and in the informal thinking subscales of Quantity Comparison (d = 0.72), Informal Calculation (d = 0.79), and Informal Calculation (d = 0.79) and Basic Informal Concepts (d = 0.63). Finally, we observed greater differences in symbolic comparison (d = 1.0), in the PI Numeracy scale (d = 0.9) and in the total informal thinking scale (d = 0.92).

Upon analyzing the differences between the correlation matrices across Spain and Chile using the Steiger Test, these differences turned out to be significant, X^2^(36) = 63.2, *p* = 0.0034. It can be observed that all the correlations were statistically significant except for the relations in Chile between keys and vocabulary (r = 0.15), informal calculation and processing speed (r = 0.23), and conceptualization and processing speed (r = 0.17) (See Table 3). In general terms, the correlations were higher in Spain than in Chile, except in 4 correlations, but where there was no statistically significant difference between the correlations of both countries in the following variables, using the Steiger Test: Vocabulary and symbolic comparison (*p* = 0.7), symbolic comparison and non-symbolic comparison (*p* = 0.95), symbolic comparison and informal calculation (*p* = 0.94), as well as between PI-conceptualization and the total scale (*p* = 0.81).

Continuing our data analysis, first of all the measurement model for Informal Thinking was tested in each country using Confirmatory Factorial Analysis. As shown in Table 4, in all of the cases the model fits the data and, therefore, both generally and by country, the instrument functions adequately.

Table 5 presents the configurational, metric and scalar invariance models. While the CFI difference between the configurational and metric invariance models exceeds the 0.01 limit, it can be observed that, in all cases, the model fits the data, with an insignificant chi-squared for each model. Aside from this, the difference between models turns out to be non-significant, using the scaled difference chi-square test statistic.

Table 6 presents the different models of predictive invariance of informal thinking. As can be observed, all the models fit the data both absolutely, with non-significant Chi-Squared tests, and with adequate relative fit indicators, CFI > 0.95 and RMSEA < 0.08. Considering that under different conditions of measurement invariance, including those where the regression coefficients are equal by country, we can affirm the predictive invariance of general and specific determinants of informal thinking.

In Table 7, it is observed that all of the factorial coefficients turned out to be significant, with the Numeration coefficient as the highest, followed by the coefficients of quantity comparison, informal calculation and basic informal concepts. All of the regression coefficients of the general and specific determinants can be observed as being significant. The age coefficients for Chile and Spain are positive and both are significant, while the female gender coefficients are negative, though not significant.

Lastly, Table 8 shows the general dominance of the predictor variables over informal thinking. It can be observed that non-symbolic and symbolic comparison is of greater importance in Chile, while non-symbolic comparison and processing speed are of more importance in Spain.

## 4. Discussion

### 4.1. Theoretical Framework and General Findings

In general terms, mathematical knowledge integrates concepts and skills. On one hand, it enables understanding the reasons or the “why” of the procedures; on the other, it allows the apprehension of procedural skills that enable addressing the “how”. Similarly, informal mathematical thinking, examined as a dependent variable in this study, refers to all those notions and procedures acquired outside the classroom setting. This factor also plays a significant role in the acquisition of formal mathematical knowledge in school (Libertus et al. 2016; Park et al. 2016). Both aspects should be thoroughly analyzed and enhanced, as their central role is recognized in the acquisition of mathematical knowledge or in the difficulties that may be encountered (Gravemeijer et al. 2017).

The cumulative research points to the existence of general and specific cognitive precursors as predictors of both early mathematical competencies, and difficulties or achievements in academic mathematical learning, particularly in relation to tasks of numeration, quantity comparison, informal calculation and concepts that define the construct of informal mathematical thinking (Bernal-Ruiz and Cerda 2024). In this study, the role of four of the above-referenced precursors is corroborated: two domain-general precursors (processing speed and receptive vocabulary), and two domain-specific precursors (symbolic comparison and non-symbolic comparison). The results clearly indicate a positive and significant relationship with early mathematical competence, specifically informal mathematical thinking, which aligns with findings from other studies (Merkley and Ansari 2016).

Regarding gender effects, our analyses revealed no significant gender differences in informal mathematical thinking across both samples. This finding aligns with research suggesting that gender differences in mathematics may emerge later in development, rather than at the preschool level examined in this study.

### 4.2. Similarities Across the Chilean and Spanish Samples

Several important similarities emerged between the Chilean and Spanish samples that support the universality of certain cognitive mechanisms underlying early mathematical development. First, the measurement model for informal mathematical thinking demonstrated adequate fit in both samples individually and globally (Table 4), confirming that the four-factor structure (numeration, numerical comparison, informal concepts, and informal calculation) is invariant across cultural contexts. This finding is significant as it supports the theoretical model with evidence of a cross-cultural nature that transcends simple empirical generalization.

The structural equation models revealed predictive invariance across both samples, with all models demonstrating adequate fit under different invariance conditions (Table 6). This indicates that a complex, coherent and analogous model can be identified by the relations between domain-general and domain-specific precursors and informal mathematical thinking in both samples. The invariance findings support the existence of common cognitive mechanisms underlying early numeracy development, regardless of sociocultural context.

Furthermore, domain-specific precursors demonstrated greater explanatory weight in both samples. Non-symbolic comparison emerged as the most important predictor in both contexts (Table 8), highlighting the fundamental role of magnitude processing abilities in early mathematical thinking. This finding aligns with specialized literature which considers that processing skills of symbolic magnitudes are built over the ability to represent a non-symbolic quantity (Hutchison et al. 2020; Merkley and Ansari 2016), and that the early years of schooling are crucial for building a base of mathematical knowledge (Siegler and Lortie-Forgues 2014).

### 4.3. Differences Between the Chilean and Spanish Samples

Despite the common underlying structure, significant differences emerged between the samples that reflect the impact of sociocultural and educational contexts. The comparative results showed expected differences in accordance with evolutionary patterns resulting from family environments of greater or lesser enrichment (Erturan and Jansen 2015). Statistically significant differences were found in nearly all examined variables, with the exception of receptive vocabulary and non-symbolic comparison, with Spanish sample students consistently showing higher performance levels.

The probable inference is that these differences stem from the distinct characteristics of the student populations in both countries. In the Chilean sample, students come from a more homogeneous and vulnerable socioeconomic population, generally attending schools that are the sole option for families due to cost and selectivity considerations. In contrast, the Spanish sample represents a more heterogeneous population, where school choice is driven by residential location and proximity criteria, with better-trained teaching faculty and public schools serving families from diverse socioeconomic backgrounds.

Specifically, the differences observed in processing speed and symbolic comparison variables may be attributed to greater involvement in child-rearing in Spanish families and enhanced teacher performance. Spain maintains higher standards for teacher hiring and more classroom supervision of teaching methods, practices that are less systematic in the Chilean context. These differences were particularly pronounced in symbolic comparison (d = 1.0), numbering tasks (d = 0.9), and total informal thinking scores (d = 0.92).

The dominance analysis revealed distinct patterns between samples. In the Spanish sample, non-symbolic comparison and processing speed were the variables with the greatest explanatory weight, followed by symbolic comparison. However, in the Chilean sample, non-symbolic comparison sustained the greater explanatory weight, followed by symbolic comparison and receptive vocabulary. This differential pattern suggests that Chilean students from disadvantaged contexts may be in a different developmental stage, where differences among students’ center on progress in vocabulary and relational logic domains as the basis for number knowledge (Cerda et al. 2011).

The presence of processing speed in the Spanish student model is probably based on the greater complexity of tasks performed in that educational context, related to mental calculation and activities oriented toward strengthening relational logic competencies with more complex numerical activities. This contrasts with the Chilean sample, whose activities fundamentally follow traditional approaches at the preschool level. The existing differences could also be attributed to the link between mathematical competence development and mastery of other cognitive skills, such as memory, which are affected by disadvantaged socioeconomic status (St. John et al. 2019).

### 4.4. Implications and Contextual Interpretations

These findings indicate that although foundational mathematical competencies rely on common cognitive mechanisms, cultural and educational contexts modulate the strength of these associations. The results point to significant differences in informal mathematical thinking as a function of socioeconomic and cultural contexts, as reflected by findings of other studies (Rittle-Johnson et al. 2017; Elliott and Bachman 2018). These differences were manifested in all components of informal mathematical thinking and in overall scores as well.

Thus, it would seem that the acquisition of symbolic skills occurs more quickly in contexts that are less vulnerable socioeconomically, and the observed differences between students according to their context may originate in the greater stimulation received and early expression of symbolic systems. Hence, disadvantaged contexts can benefit from early interventions designed to buffer the creation and widening of the socioeconomic gap (Scalise et al. 2018).

In the Chilean sample, the presence of vocabulary as a significant predictor may be interpreted as resulting from greater lexical availability in Spanish curriculum orientation toward the reading process at an earlier age. This aligns with the existence of a possible context-based vocabulary gap (Coddington et al. 2014). Informal mathematical thinking presents itself as a fundamental pillar for laying the groundwork of formal knowledge from the start of schooling. Informal experiences in the family environment therefore contribute to learning, and as a result, a stimulating context can favor improved development of numeracy skills, exceeding the level reached at the beginning of formal education by children growing up in disadvantaged contexts (Elliott and Bachman 2018).

### 4.5. Relationship Between Hypotheses and Results

Regarding our initial hypotheses, the results provide clear support for both proposed predictions. Hypothesis 1, which anticipated significant differences in informal mathematical thinking favoring the Spanish sample due to contextual differences and early stimulation, was confirmed across nearly all examined variables. As shown in Table 2, significant differences emerged in processing speed (d = 0.66), symbolic comparison (d = 1.0), numbering (d = 0.9), numerical comparison (d = 0.72), informal concepts (d = 0.79), informal calculation (d = 0.63), and total informal thinking scores (d = 0.92), all favoring the Spanish sample.

Hypothesis 2, proposing that a robust SEM framework could identify interactions between general and domain-specific cognitive precursors and their explanatory contribution to informal mathematical thinking, was also supported. The structural equation models demonstrated adequate fit across all invariance conditions (Table 6), with significant contributions from both domain-general predictors (receptive vocabulary and processing speed) and domain-specific predictors (symbolic and non-symbolic comparison). The dominance analysis (Table 8) revealed that domain-specific precursors had greater explanatory weight in both samples, though with different patterns: non-symbolic comparison dominated in both contexts, while symbolic comparison was more prominent in the Chilean sample and processing speed in the Spanish sample.

## 5. Conclusions and Implications

The above differences between the Chilean and Spanish groups support the need to set in motion education strategies or programs of interventions that will aid in the mitigation of these existing, context-related differences (Eason et al. 2020; Hascoët et al. 2021). Using natural situations for mathematical learning, from the starting point of informal mathematical knowledge, is an opportunity to seed processes for the development of early mathematical skills, such as counting, subitizing, conceptual apprehension of shapes and measures, etc. Early recognition of the importance and role of domain-general and domain-specific precursors, such as those analyzed in this comparative study, presents itself as a preventive mechanism against the learning difficulties that students in the Spain sample or Chilean sample may encounter. The reported findings on the sound role of general and specific cognitive skills, their relationship with formal mathematical thinking observed in the students of both countries, constitute without a doubt a significant contribution to the prevention of long-term learning difficulties in mathematics and to enabling interventions in early childhood education with a high probability of success, as other studies have concomitantly put forward (Peres Nogues and Vargas Dorneles 2021).

While these contributions are certainly important, they also prompt further scientific questions, the resolution of which is challenging due to the limitations inherent in the study. Firstly, the non-probabilistic sampling approach and the sample size limitations do not allow generalization of the results to the entire preschool populations, though they provide valuable insights into the cognitive architecture of informal mathematical thinking in these specific educational contexts to present evidence that opens up spaces of inquiry into the barriers to social mobility that are deeply ingrained in the economic, educational and societal structures evidenced in both countries (Segura-Carrillo 2025).

Secondly, the absence of a more specific measure of family SES than the traditional association of SES based on the type of administration that the establishment has, and which, while supported by the literature, does not enable adequately capturing the specificity of the groups compared, is another limitation of the study and should be included in future research.

Aside from the above, yet another limitation is that, although the groups from the two countries are of more or less the same age, they all attend public schools and were assessed using the same instruments and measurement procedures, the marked social stratification in the Chilean system may constitute a significant bias. This is especially true in light of a study conducted in Chile that found that six and seven year olds from a high-income socioeconomic background consistently scored higher in math and reading tasks and in measures of executive functions, compared to their peers from a lower-income socioeconomic background (Escobar et al. 2018). On the other hand, the role of the students’ parents and families was unexamined—their practices, beliefs or perceptions of mathematical competence, considering that some of these factors have a significant correlation to socioeconomic level in terms of their frequency and pertinence (Douglas et al. 2021).

Despite these limitations, this study yields several conclusions pertinent to the educational contexts of both countries. First of all, the informal mathematical knowledge that is acquired outside the school context plays an essential part in the acquisition of formal academic mathematical knowledge. Thus, it is essential to analyze and promote the understanding of mathematical concepts, as well as the learning of procedural skills in order to improve mathematical performance. Secondly, there are general and specific cognitive precursors that predict early mathematical competencies and success or difficulties in academic mathematical learning. Processing speed, receptive vocabulary, symbolic and non-symbolic comparison were significant precursors of this relationship. Finally, statistically significant differences in mathematical performance were observed among students from differing socioeconomic contexts in the samples studied. Students from more advantageous backgrounds tend to perform better in various math areas and in relation to different precursors. Hence, these differences in mathematical performance among different socioeconomic contexts underscore a need for the implementation of education strategies and intervention programs to address these disparities and promote an equitable development of mathematical skills.

## Figures and Tables

**Table 1:** Sample distribution by country, gender and age (in months).

Country	Gender (Age)		Total
	Girls (M; SD)	Boys (M; SD)	
Chile	33 (53.00; 2.09)	32 (53.09; 2.24)	65 (53.05; 2.15)
Spain	30 (54.77; 2.47)	35 (55.43; 2.62)	65 (55.12; 2.55)
Total	63 (53.84; 2.43)	67 (54.31; 2.70)	130 (54.08; 2.57)

**Table 2:** Descriptive data for total and subscale scores of informal thinking, as well as general and specific determinants, for both Spain and Chile.

	Total		Chile		Spain.		Welch’s *t* Test		
Variables	M	SD	M	SD	M	SD	Statistical	*p* Value	*ES*(*d*)
Receptive Vocabulary.	11.02	2155	10.69	2.172	11.35	2.102	*t*(127.9) = 1.76	0.08	0.31
Processing Speed.	18.35	11.48	14.74	9.082	21.97	12.51	*t*(116.8) = 3.77	<0.001	0.66
Symbolic Comparison.	20.93	11.85	15.60	10.12	26.26	11.08	*t*(127.0) = 5.73	<0.001	1.0
Non-symbolic Comparison.	25.85	12.37	23.94	11.21	27.75	13.24	*t*(124.6) = 1.77	0.079	0.31
Numbering.	7.969	3.777	6.415	2.384	9.523	4.261	*t*(100.5) = 5.13	<0.001	0.9
Numerical Comparison.	1.615	0.9991	1.277	0.696	1.954	1.138	*t*(106.0) = 4.09	<0.001	0.72
Informal Concepts.	1.308	1.299	0.831	0.945	1.785	1.431	*t*(110.9) = 4.49	<0.001	0.79
Informal Calculation.	1.123	0.8628	0.862	0.827	1.385	0.823	*t*(128.0) = 3.61	<0.001	0.63
Informal Thinking.	12.03	6.306	9.385	4.069	14.68	7.027	*t*(102.6) = 5.25	<0.001	0.92

**Table 3:** Pearson’s Matrix of Correlations between the Scales in Chile and Spain.

	Receptive Vocabulary.		Processing Speed.		Symbolic Comparison.		Non-Symbolic Comparison.		Numbering.		Numerical Comparison.		Informal Concepts.		Calculation.		Informal Thinking.	
Receptive Vocabulary.	1	**	0.15		0.35	**	0.4	**	0.39	**	0.35	**	0.39	**	0.35	**	0.45	**
Processing Speed.	0.38	**	1	**	0.22		0.35	**	0.47	**	0.26	*	0.23		0.17		0.41	**
Symbolic Comparison.	0.43	**	0.53	**	1	**	0.35	**	0.41	**	0.44	**	0.53	**	0.42	**	0.52	**
Non-Symbolic Comparison.	0.34	**	0.61	**	0.34	**	1	**	0.42	**	0.36	**	0.42	**	0.41	**	0.49	**
Numbering.	0.48	**	0.59	**	0.5	**	0.64	**	1	**	0.51	**	0.59	**	0.61	**	0.94	**
Numerical Comparison.	0.45	**	0.46	**	0.44	**	0.49	**	0.78	**	1	**	0.57	**	0.42	**	0.69	**
Calculation	0.54	**	0.47	**	0.52	**	0.54	**	0.8	**	0.74	**	1	**	0.51	**	0.78	**
Informal Concepts	0.42	**	0.47	**	0.52	**	0.63	**	0.66	**	0.55	**	0.6	**	1	**	0.75	**
Informal Thinking.	0.52	**	0.58	**	0.54	**	0.66	**	0.98	**	0.85	**	0.89	**	0.73	**	1	**

** *p* < 0.01, * *p* < 0.05.

**Table 4:** Confirmatory Factorial Analysis for all cases, Chile and Spain.

Model	χ^2^	χ^2^/gl	CFI	TLI	SRMR	RMSEA
Global Model	*χ*^2^(2) = 2.77, *p* = 0.251	1384	0997	0991	0016	0.054 [0.000, 0.193], *p* = 0.356
Chile Model	*χ*^2^(2) = 2.37, *p* = 0.306	1.186	0.995	0.984	0.031	0.053 [0.000, 0.246], *p* = 0.370
Spain Model	*χ*^2^(2) = 1.08, *p* = 0.581	0.542	1	1.018	0.013	0.000 [0.000, 0.220], *p* = 0.605

**Table 5:** Measurement Invariance Models for Informal Thinking Across Spain and Chile.

Invariance Model	χ^2^	χ^2^/gl	CFI	TLI	SRMR	RMSEA	Diff CFI	Diff χ^2^
Configurational Invariance	χ^2^(4) = 3.62, *p* = 0.460	0.904	1	1.005	0.019	0.000 [0.000, 0.179], *p* = 0.543	NA	NA
Metric Invariance	χ^2^(7) = 11.71, *p* = 0.110	1.673	0.978	0.963	0.084	0.102 [0.000, 0.197], *p* = 0.178	−0.022	χ^2^(3) = 7.01, *p* = 0.072
Scalar Invariance	χ^2^(10) = 12.10, *p* = 0.278	1.21	0.99	0.988	0.086	0.057 [0.000, 0.149], *p* = 0.407	0.012	χ^2^(3) = 0.48, *p* = 0.924

**Table 6:** Predictive Invariance Models for Informal Thinking across Spain and Chile.

Models	χ^2^	χ^2^/gl	CFI	TLI	SRMR	RMSEA
Configurational Invariance—Unconstrained Regression Coefficients	χ^2^(40) = 48.24, *p* = 0.174	1.206	0.977	0.966	0.038	0.056 [0.000, 0.109], *p* = 0.406
Scalar Invariance—Unconstrained Regression Coefficients	χ^2^(47) = 57.32, *p* = 0.144	1.219	0.972	0.964	0.057	0.058 [0.000, 0.106], *p* = 0.382
Configurational Invariance—Regression Coefficients Constrained to be Equal Across Countries	χ^2^(44) = 59.86, *p* = 0.056	1.36	0.956	0.94	0.071	0.074 [0.000, 0.120], *p* = 0.208
Scalar Invariance—Regression Coefficients Constrained to be Equal Across Countries	χ^2^(51) = 68.00, *p* = 0.056	1.333	0.953	0.945	0.09	0.072 [0.000, 0.114], *p* = 0.221

**Table 7:** Structural Equation Model (SEM) Coefficients for Predictive Invariance of Informal Thinking.

	Estimator	*p* Value
Factorial coefficients		
Numbering	1	--
Numerical Comparison	0.2361	<.001
Informal Concepts	0.3264	<.001
Informal Calculation	0.1766	<.001
Regression coefficients		
Receptive Vocabulary.	0.1968	0.0296
Processing Speed	0.05439	0.0190
Symbolic Comparison	0.07481	0.0001
Non-Symbolic Comparison	0.0683	<0.001
Age (Chile)	0.2005	0.0372
Females (Chile)	−0.6231	0.1604
Age (Spain)	0.2134	0.0219
Females (Spain)	−0.009492	0.9888

**Table 8:** General Dominance of Predictor Variables of Informal Thinking.

	Chile	Spain
Non-Symbolic Comparison	0.188	0.134
Symbolic Comparison	0.183	0.116
Vocabulary	0.122	0.066
Processing Speed	0.094	0.134
Sociodemographic	0.099	0.076
R^2^ total	0.686	0.526

## Data Availability

The data presented in this study are available on request from the corresponding author due to confidentiality agreements with participating students that restrict data sharing beyond the original research team. Any data sharing is subject to their prior authorization.
